# Systematic Review and Meta-Analysis of the Variants of the Obturatory Artery

**DOI:** 10.3390/jcm12154932

**Published:** 2023-07-27

**Authors:** Gioia Brachini, Matteo Matteucci, Paolo Sapienza, Roberto Cirocchi, Alessandro Favilli, Stefano Avenia, Isaac Cheruiyot, Giovanni Tebala, Piergiorgio Fedeli, Justin Davies, Justus Randolph, Bruno Cirillo

**Affiliations:** 1 Department of Surgery, Sapienza University, Viale del Policlinico 155, 00161 Rome, Italy; gioia.brachini@uniroma1.it (G.B.); paolo.sapienza@uniroma1.it (P.S.); 2 Department of Medicine and Surgery, University of Milan, 20122 Milan, Italy; matteo.matteucci@unimi.it; 3 Department of Medicine and Surgery, University of Perugia, 06132 Perugia, Italy; roberto.cirocchi@unipg.it (R.C.); alessandro.favilli@unipg.it (A.F.); stefanoavenia1@gmail.com (S.A.); 4 Department of Human Anatomy, University of Nairobi, P.O. Box 30197, Nairobi 00100, Kenya; isaacbmn@outlook.com; 5 Department of Surgery, Azienda Ospedaliera Santa Maria di Terni, 05100 Terni, Italy; gtebala@gmail.com; 6 Department of Legal Medicine, University of Camerino, 62032 Camerino, Italy; piergiorgio.fedeli@unicam.it; 7 Cambridge Colorectal Unit, Addenbrooke’s Hospital, University of Cambridge, Cambridge CB0QQ, UK; justin.davies7@nhs.net; 8 Georgia Baptist College of Nursing, Mercer University, Atlanta, GA 30341, USA; randolph_jj@mercer.edu

**Keywords:** obturator artery (OA), internal iliac artery (IIA), external iliac artery (EIA)

## Abstract

Background: Knowledge of vascular anatomy and its possible variations is essential for performing embolization or revascularization procedures and complex surgery in the pelvis. The obturator artery (OA) is a branch of the anterior division of the internal iliac artery (IIA), and it has the highest frequency of variation among branches of the internal iliac artery. Possible anomalies of the origin of the obturator artery (OA) should be known when performing pelvic and groin surgery, where its control or ligation may be required. The purpose of this systematic review and meta-analysis, based on Sanudo’s classification, is to analyze the origin of the obturator artery (OA) and its variants. Methods: Thirteen articles published between 1952 and 2020 were included. Results: The obturator artery (OA) was present in almost all cases (99.8%): the pooled prevalence estimate for the origin from the IIA axis was 77.7% (95% CI 71.8–83.1%) vs. 22.3% (95% CI 16.9–28.2%) for the origin from EIA axis. In most cases, the obturator artery (OA) originated from the anterior division trunk of the internal iliac artery (IIA) (61.6%). Conclusions: Performing preoperative radiological examination to determine the pelvic vascular pattern and having the awareness to evaluate possible changes in the obturator artery can reduce the risk of iatrogenic injury and complications.

## 1. Introduction

The Obturator Artery (OA) is an extrapelvic parietal branch of the Internal Iliac Artery (IIA) that supplies the medial thigh muscles. The OA runs forward and down on the lateral wall of the small pelvis, subsequently exiting through the obturator canal to enter the medial compartment of the thigh. After the obturator foramen, the OA divides into its anterior and posterior branches.

In common clinical practice, the following OA variations may be commented on when:

- The OA is aberrant when it does not originate from the anterior division of the IIA but originates from the External Iliac Artery (EIA) axis.
- The OA is an accessory when there is a double OA, either branching from the IIA or EIA axis.

In 2011, Sanudo et al. [1] standardized the classification of OA variations, in which six different types were reported. In Type A, the OA originates from the anterior division of the IIA; in Type B, the artery originates from the Inferior Epigastric Artery (IEA). These two types of variations are also the most common. In Type C, the origin is at the level of the posterior division of the IIA, while in Type D, the artery derives from the IIA above its final branching. Finally, the rarest cases are distinguished as Type E, when the artery derives from the EIA, and Type F, where it arises from the femoral artery.

It is essential for physicians to be familiar with the anatomical variations of OA when performing invasive procedures involving the pelvis. Surgeons should be aware of the course of the arteries and their potential different paths to prevent undesirable and potentially life-threatening complications.

Although the anatomical variations of the OA are well documented according to Sanudo’s classification [1], there is still a lack of a global estimation regarding their prevalence in the scientific literature. Considering these elements, the aim of this systematic review and meta-analysis was to determine the prevalence of the OA and its anatomical variants according to Sanudo’s [1] classification.

## 2. Materials and Methods

A systematic review and meta-analysis were performed according to the guidelines set out in “the Preferred Reporting Items for Systematic Reviews and Meta-Analyses (PRISMA) guidelines”.

### 2.1. Research Strategies

This systematic search, updated to 1 January 2023, was performed using PubMed, SCOPUS, and Web of Science (WOS). PubMed terms used for the search were the following: “Obturator Artery” and “variants”. No language restrictions were made.

The research has been expanded with “Google Scholar” and Pubmed’s function “related articles”.

### 2.2. Selection Criteria: Inclusion and Exclusion Criteria

In order to include a study in the meta-analysis, it needed to meet the following criterion: a prevalence of reported data on the origin of the OA. All case reports, editorials, letters, reviews, and studies with irrelevant or incomplete data were excluded from the study.

### 2.3. Data Extraction

Two authors (M.M. and R.C.) extracted data from the included studies, including the surname of the first author of the study, the year of publication, the country of origin of the hospital where the study was carried out, the type of study and the number of cadavers included.

### 2.4. Statistical Methods

In addition to calculating raw prevalence, we used MetaXL (Version 5.3) to estimate multinomial pooled prevalence estimates (PPEs) and their 95% CIs using a DerSimonian and Laird random effects model with double arcsine transformations. Multinomial pooled prevalence estimates were normalized such that the summated prevalences equaled 1.0. Heterogeneity was examined statistically through the I^2^ statistic and LFK_index_. Barendregt and Doi’s (2016) LFK_index_ is an effect-size-like statistic for measuring the degree of asymmetry in a funnel-type display of study effects. We used the standard criteria reported by Higgins et al. (2021) for judging heterogeneity via the I^2^ statistic. In the lesser-known LFK_index_, a value of 0.00 indicates symmetry, values between 0.00 and ±2.00 indicate minor asymmetry, and values greater than ±2.00 indicate major asymmetry (Barendregt and Doi, 2016). Heterogeneity was examined visually with forest plots, Doi plots, and funnel plots. To examine the sensitivity of models to individual study outliers, we conducted a leave-one-out analysis.

### 2.5. Risk of Bias Assessment of the Studies Included

Quality assessment and bias risk analysis of all selected full-text articles were performed using the International Evidence-Based Anatomy (iEBA) Group’s Anatomical Quality Assurance (AQUA) tool [Henry et al., 2016]. Two of the authors (R.C. and M.M.) screened the articles and assessed the risk of bias according to the five domains used in the AQUA tool. In the case of a discordant evaluation, a third author (S.A.) was involved in reaching a consensus.

## 3. Results of Systematic Research

### 3.1. Identification of Studies

**Figure 1:**
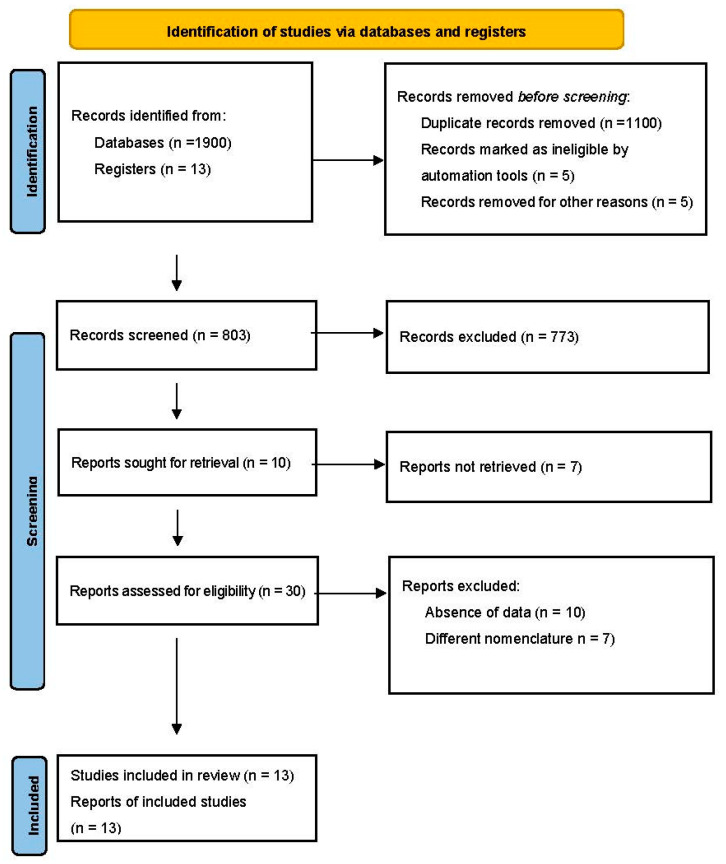
Prisma flow chart of literature search.

An initial search yielded 1900 potentially relevant articles and 147 articles from additional documents identified through other sources (grey literature). After removing duplicates and analyzing remaining titles and abstracts, further articles were excluded, leaving 30 studies for full-text analysis. Of these, 17 were excluded due to the absence of data reported in the article or to a different terminology of arterial vessels. Therefore, 13 articles were included in this systematic review and meta-analysis (Figure 1).

### 3.2. Features of the Included Studies

Thirteen articles (N = 561 cadavers) published between 1952 and 2020 were included (Table 1).

In our review, studies on radiological investigations or intraoperative findings were excluded as they were often case reports. In addition, some studies based on cadaveric dissections were also excluded as only case reports were presented, and it was not possible to establish the prevalence of the origin of the OA. Geographically, most studies were conducted in Asia (9 studies: 282 cadavers, 50.3%) [2,3,4,5,6,7,8,9,10] only a few studies were performed in Europe (3 studies: 261 cadavers, 46.5%) (Al-Talalwah 2016, Sañudo 2011, Braithwaite 1952) and in North America (1 study: 18 cadavers, 3.2%) (Granite 2020). No studies were carried out in Africa or Australasia. In Asia, the majority of studies were conducted in India (247 cadavers, 44%) (Kumar 2019, Verma 2016, Sakthivel 2015, Rajive 2015, Sumathilatha 2013, Biswas 2010, Pai 2009), only 1 study in Korea (18 cadavers, 3.2%) (Lee 2013) and 1 study in Malaysia (17 cadavers, 3.0%) (Jusoh 2010) In Europe, the studies were carried out in the United Kingdom only and in North America in the USA.

**Figure 2:**
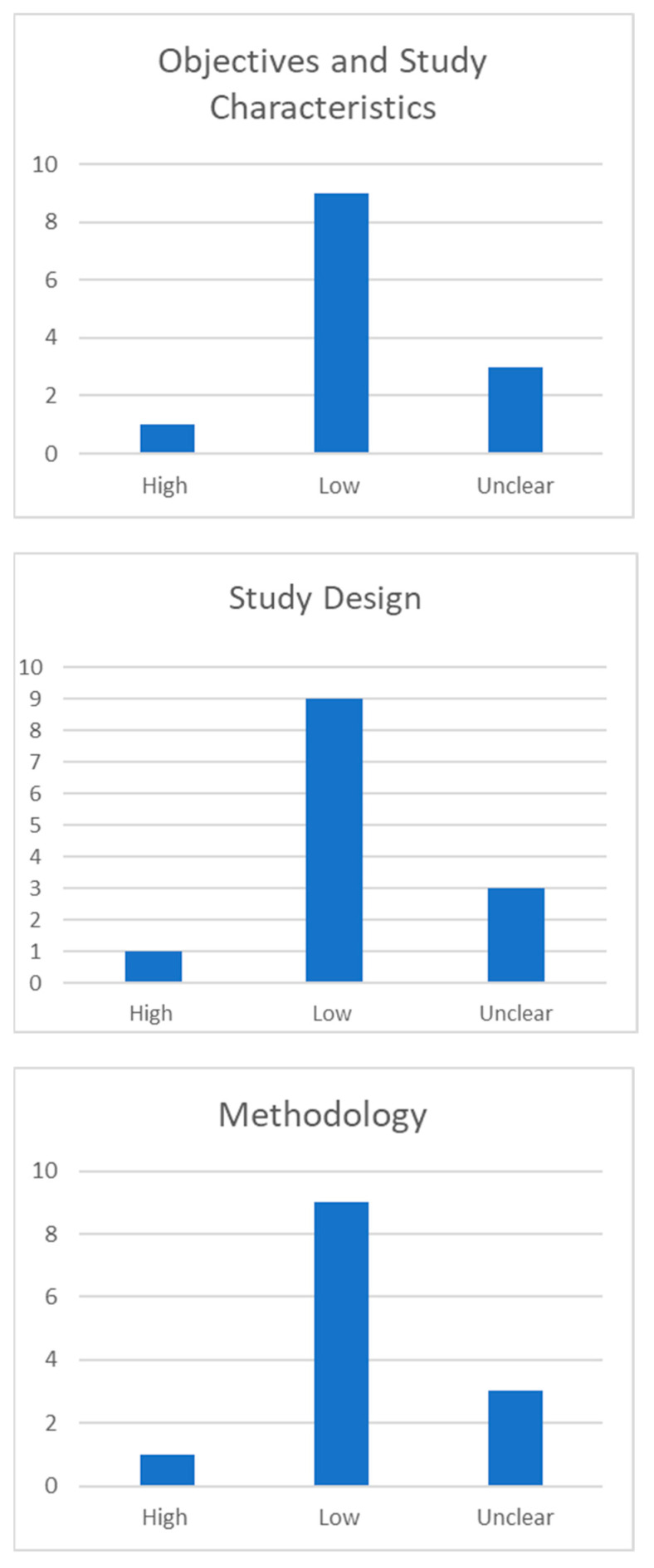

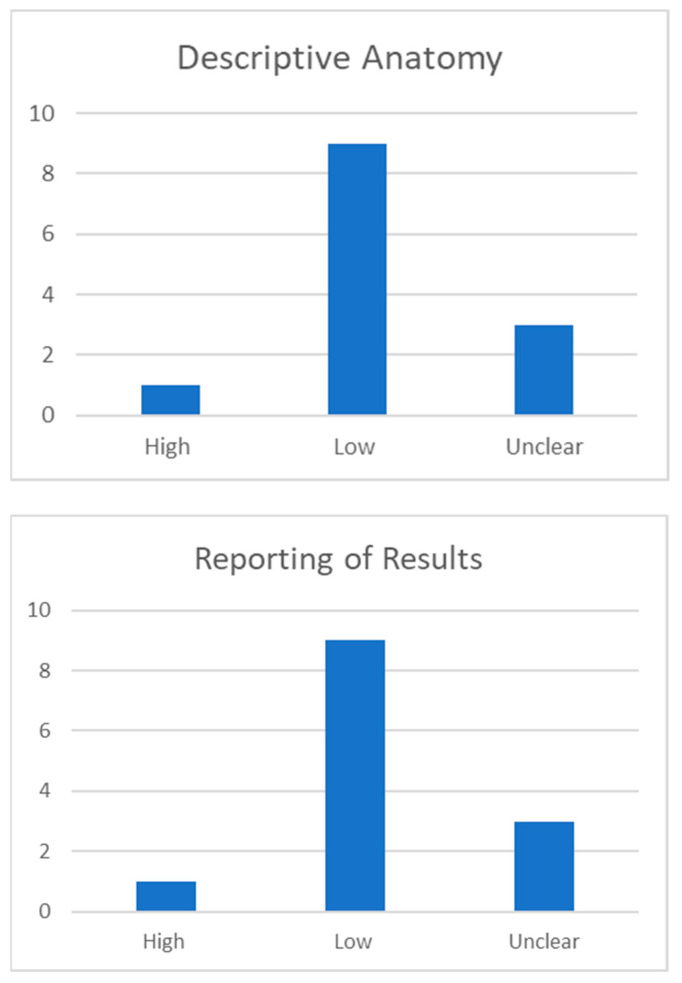
Summary Chart Quality and risk of bias assessment as determined by the AQUA tool. The risk of bias in each domain is normally categorized as “low”, “high”, or “unclear”. Five different domains are taken into consideration: objectives and study characteristics, study design, methodology characterization, descriptive anatomy, and reporting of results.

The AQUA tool probes for potential risk of bias in 13 study domains (objectives and subject characteristics, study design, methodology characterization, descriptive anatomy, and reporting of results). The risk of bias within each domain is normally categorized as “Low”, “High”, or “Unclear”. Three of the included studies showed high risk in domain one (Objective and Study Characteristics), mainly because the methods applied in them were not described enough detail to be reproduced. Similarly, one study had high risk of bias in domains two (objective and subjective characteristics), three (methodology), and five (reporting of results). A summary of the assessment of quality and risk of bias by the AQUA tool is displayed below (Figure 2).

### 3.3. Analysis of the Results of the Systematic Review

#### 3.3.1. Main Outcomes

Origin of OA. In all studies, the presence of OA was revealed in 99.8% (560 cadavers), and it was absent in only 1 case (0.2%) [11]. In all cases, the OA originated from the IIA or EIA, but the origin from the Femoral Artery was never recorded, as evidenced in the previous revision of Sañudo (1.7%) [1]. In a minority of cases, the OA was double in 15 cadavers (2.7%) [1,7,10,11,12,13], and 1 cadaver had triple arteries (0.2%) [1], consisting of an upper, middle, and lower branch.

In the cadaveric dissections, the most common origin of the OA was from the IIA (813 hemipelvis, 74.4%); in the remaining cadaveric dissections (279 hemipelvis, 25.6%), the origin of the OA was from the EIA (Table 2). Note that the percentages reported in these last two paragraphs are raw percentages; see the next section for the proportions (called pooled prevalence estimates) using a meta-analytic synthesis approach.

**Figure 3:**
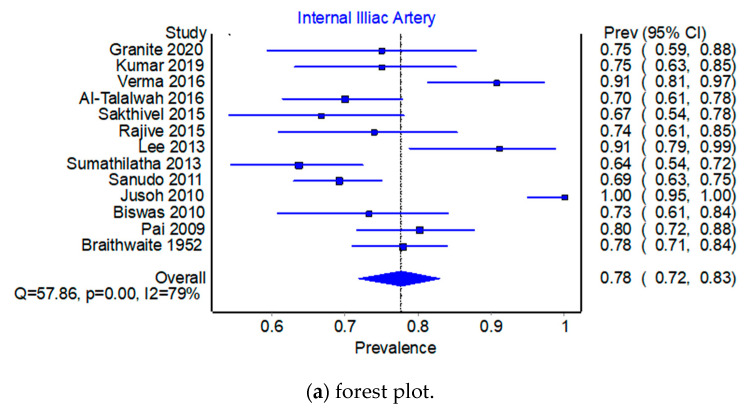

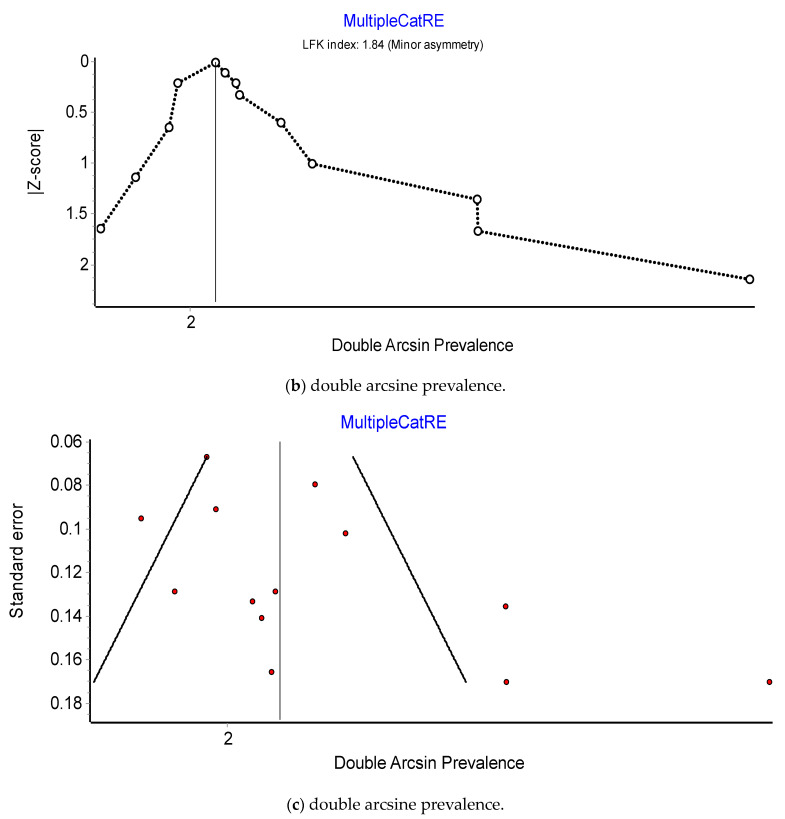
Prevalence from the Internal Iliac Artery.

**Figure 4:**
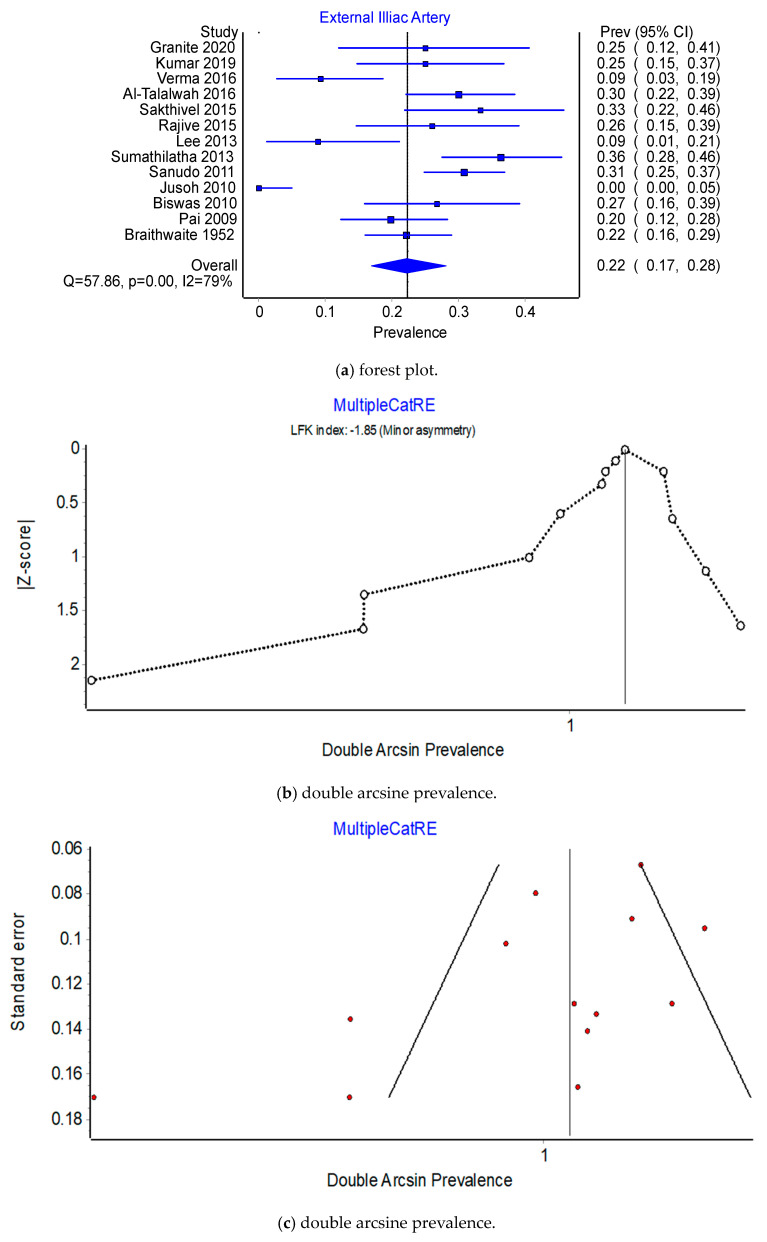
Prevalence of the External Iliac Artery.

Pooled Prevalence Estimates of origin of OA from IIA versus EIA. Across the 13 studies that reported on the prevalence of internal iliac artery (IIA) versus the external iliac artery (EIA) origin, the pooled prevalence estimate (PPE) for IIA was 77.7% (95% CI 71.8–83.1%) compared to 22.3% (95% CI 16.9–28.2%) for the EIA. There was substantial heterogeneity for this finding; I^2^ = 79.3% (95% CI 65.2–87.6%) (see the forest plots in Figure 1a and Figure 2a), Q(1) = 57.86, *p* < 0.001; and there was evidence of minor asymmetry with an LFK_index_ of ±1.85. Leave-one-out PPEs ranged from 75.0% to 78.9% for the IIA (Figure 3b,c) and 21.1% to 25.0% for the EIA (Figure 4b,c). The Jusoh 2010 study appeared to be an outlier with a reported prevalence of 100.0% (95% CI 95.0–100.0%) for the EIA; this study is based on 34 samples only, and this the small-number bias is present.

#### 3.3.2. Secondary Outcomes

**Figure 5:**
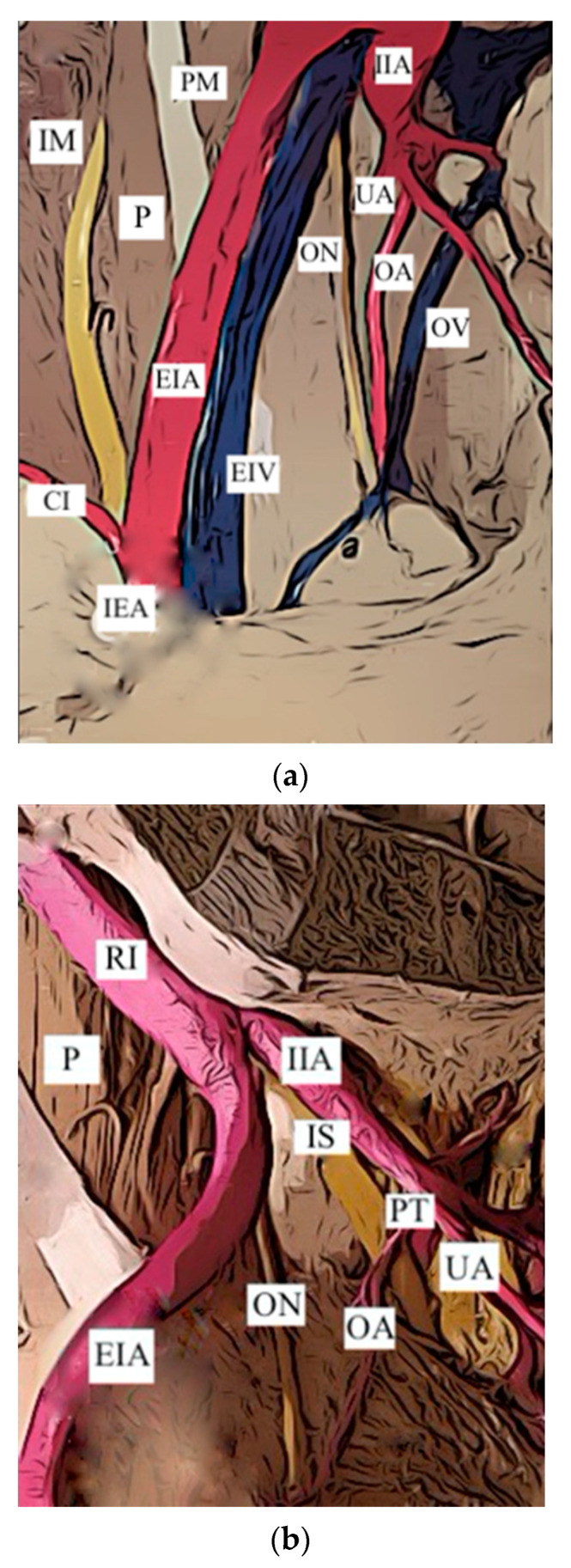

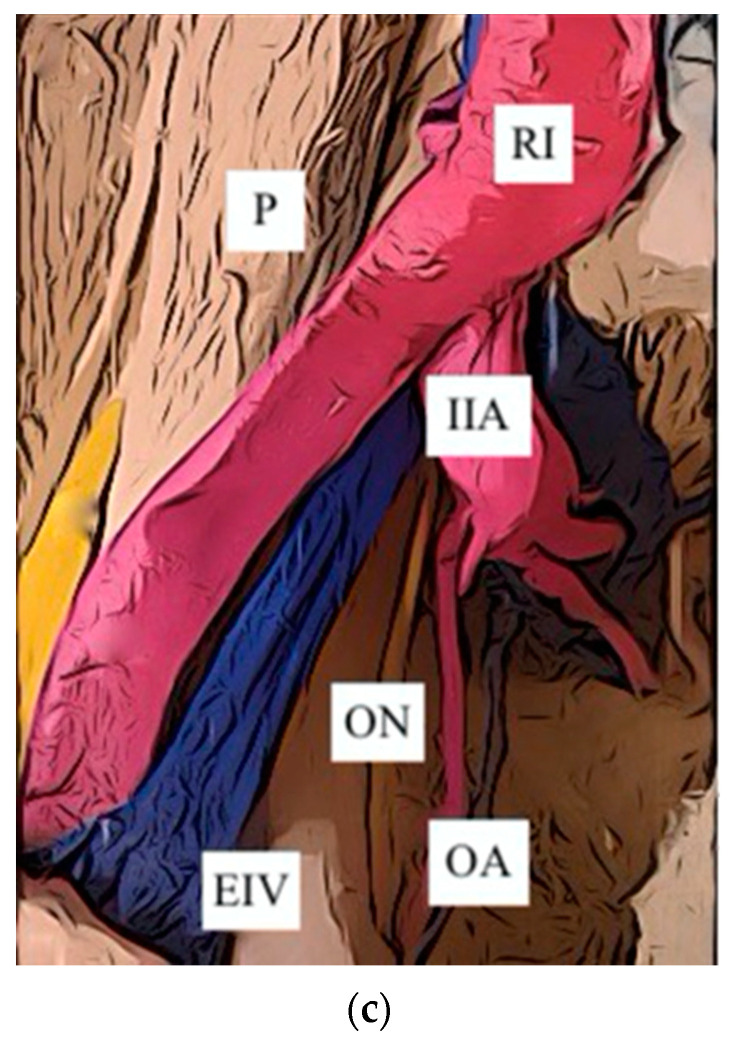
(**a**) Type A. Obturator artery arising from the anterior trunk of the internal iliac artery. CI: circumflex iliac artery; EIA: external iliac artery; EIV: external iliac vein; IIA: internal iliac artery; IEA: inferior epigastric artery; IM: iliac muscle; OA: obturator artery; ON: obturator nerve; OV: obturator vein. (**b**) Type C. Obturator artery arising from the posterior trunk of the internal iliac artery. EIA: external iliac artery; IIA: internal iliac artery; OA: obturator artery; ON: obturator nerve; P: psoas muscle; PT: posterior trunk of the internal iliac artery; RI: right iliac artery; UA: umbilical artery. (**c**) Type D. Obturator artery arising from the internal iliac artery, main trunk. EIV: external iliac vein; IIA: internal iliac artery; OA: obturator artery; ON: obturator nerve; P: psoas muscle; RI: right iliac artery.

Origin of the OA from IIA. The origin of the OA from the IIA has been divided into three subgroups, already identified in the review of Sañudo:

- Sañudo A (Figure 5a): origin from the anterior division trunk of the IIA (482 hem—pelvis, 61.6%). In the literature, this variant represents this condition and is consid—ered as the “normal anatomy”.
- Sañudo C (Figure 5b): origin from the posterior division trunk of the IIA (251 hem—pelvis, 32.1%) (Table 3). The limitation of the latter classification proposed by Sañudo is related to the fact that many vessels were included in the posterior trunk (Sañudo C), even if they had a separate origin.
- Sañudo D (Figure 5c): origin from the main trunk of the IIA (22 hemipelvis, 2.8%).

**The new analysis of the origin of the OA shows us the following prevalence:**

- origin from the main trunk of the IIA (22 hemipelvis, 2.8%)
- origin from the anterior division trunk of the IIA (482 hemipelvis, 61.6%)
- origin from the posterior common trunk cmor
- (174 hemipelvis, 22.3%)
- origin from the superior gluteal artery (65 hemipelvis, 8.3%)
- origin from the inferior gluteal artery (23 hemipelvis, 2.9%)
- origin from the iliolumbar artery (8 hemipelvis, 1%)
- origin from the internal pudendal artery (7 hemipelvis, 0.9%)
- origin from the inferior vesicle artery (1 hemipelvis, 0.1%)

**Figure 6:**
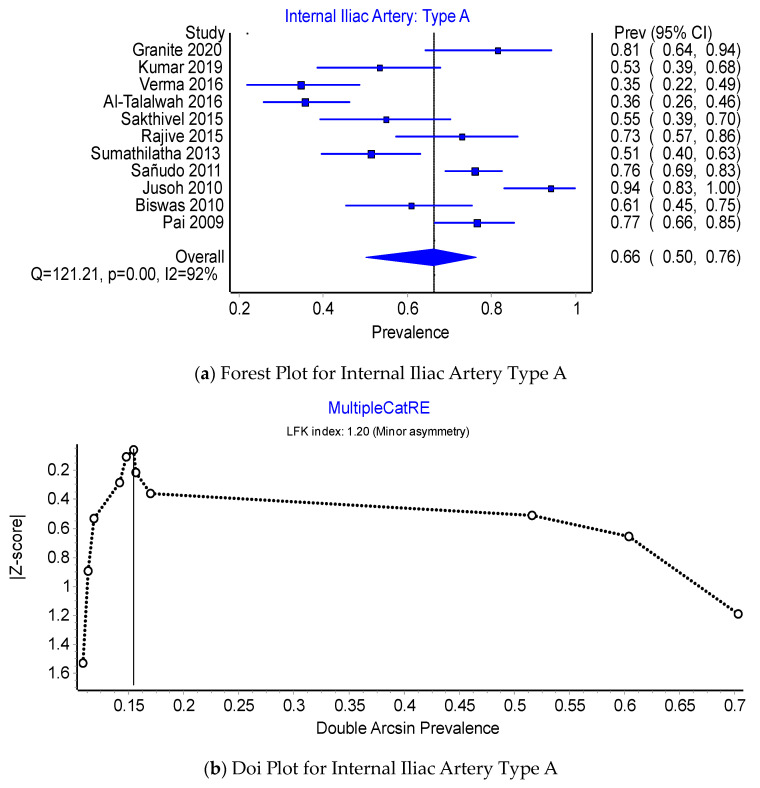

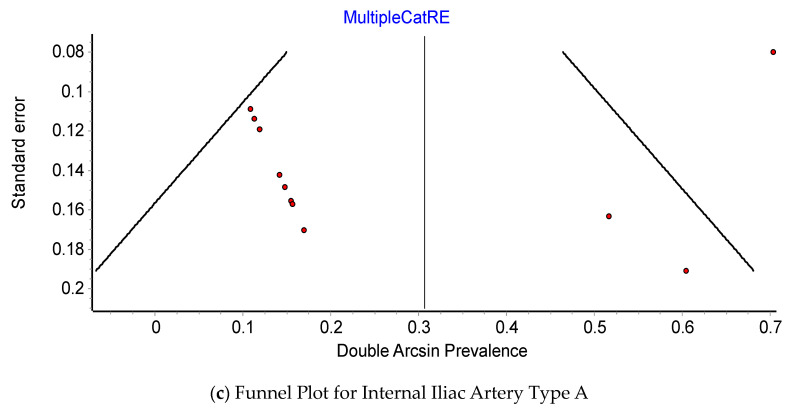
Prevalence of the Internal Iliac Artery: Type A.

**Figure 7:**
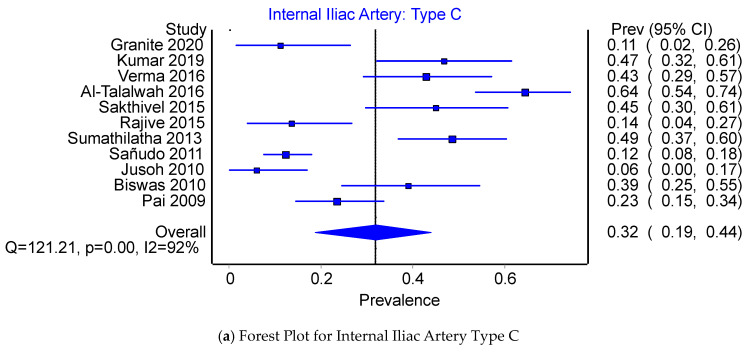

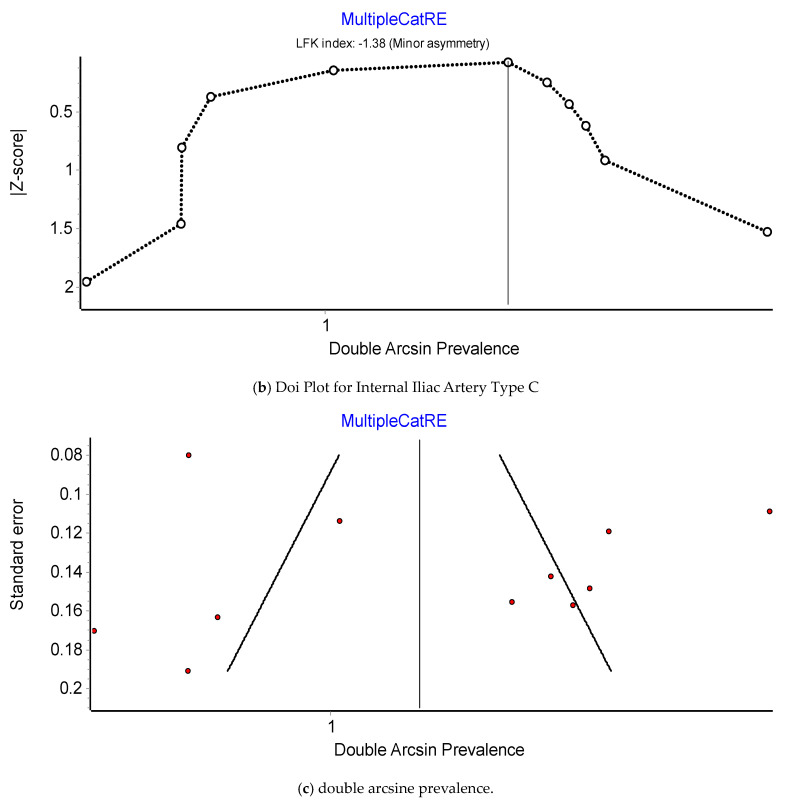
Funnel plot for Internal Iliac Artery: Type C.

**Figure 8:**
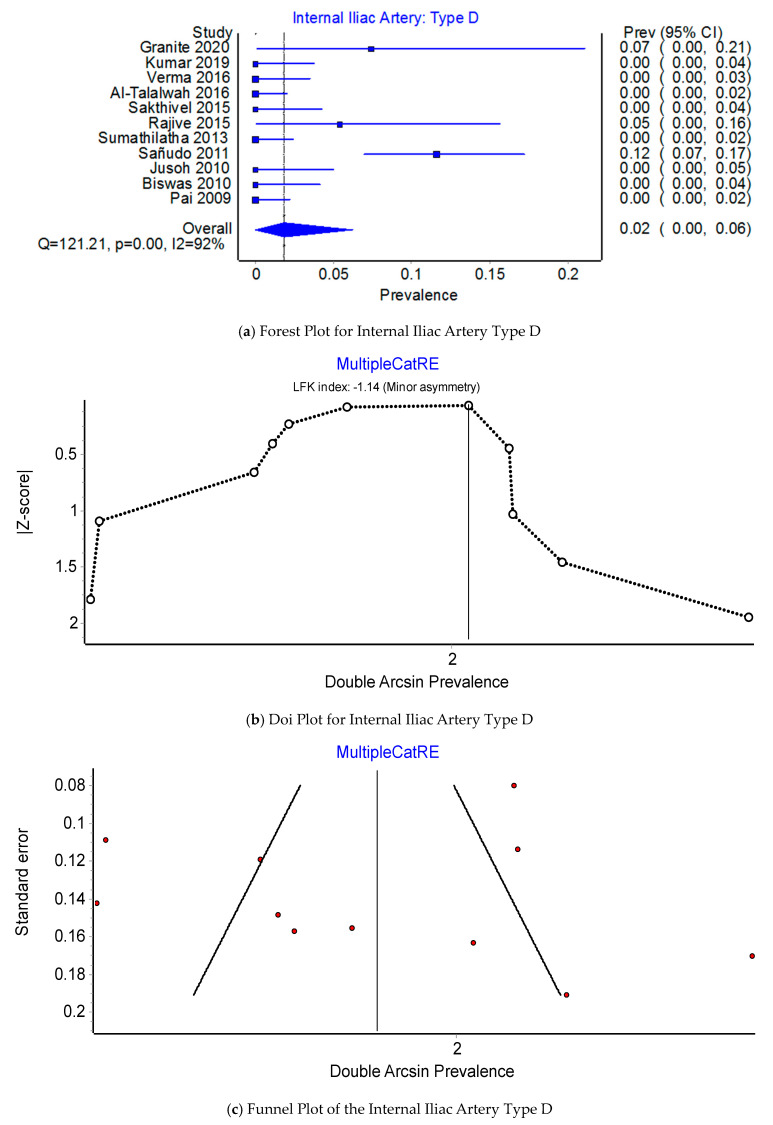
Prevalence of the Internal Iliac Artery: Type D.

Pooled Prevalence Estimates of the origin of the IIA: Type A, Type C, and Type D. Of the PPEs for Type A, Type C, and Type D origins of the IIA, 66.3% (95% CI 50.1–94.7%) were of Type A origin, 31.8% (95% CI 18.8–44.0%) were of Type C origin, and 1.8% (95% CI 0.0–6.3%) were of Type D origin. There was a considerable degree of heterogeneity across the 11 studies included in this comparison; I^2^ = 91.8% (95% CI 0.0–6.3%), Q(2) = 121.21, *p* < 0.001; and minor asymmetry, with LFK_indices_ ranging between −1.38 and 1.20. The leave-one-out range of PPEs was 62.9–69.3%, 28.7–35.1%, and 2.1–1.4% for IIA Type A (see Figure 6b,c), Type C (see Figure 7b,c), and Type D (see Figure 8b,c), respectively.

**Figure 9:**
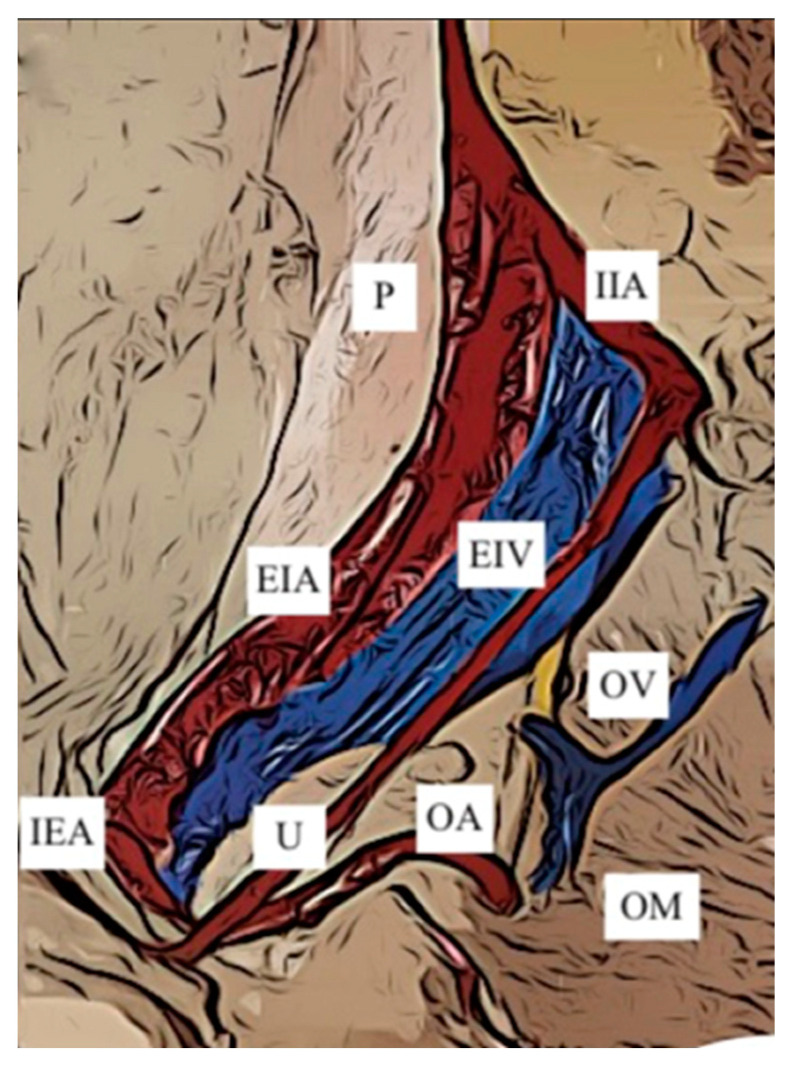
Type B. Obturator artery arising from the inferior epigastric artery. EIA: external iliac artery; EIV: external iliac vein; IIA: internal iliac artery; IEA: inferior epigastric artery; OA: obturator artery; OM: obturator muscle; OV: obturator vein; P: psoas muscle; U: umbilical artery.

**Figure 10:**
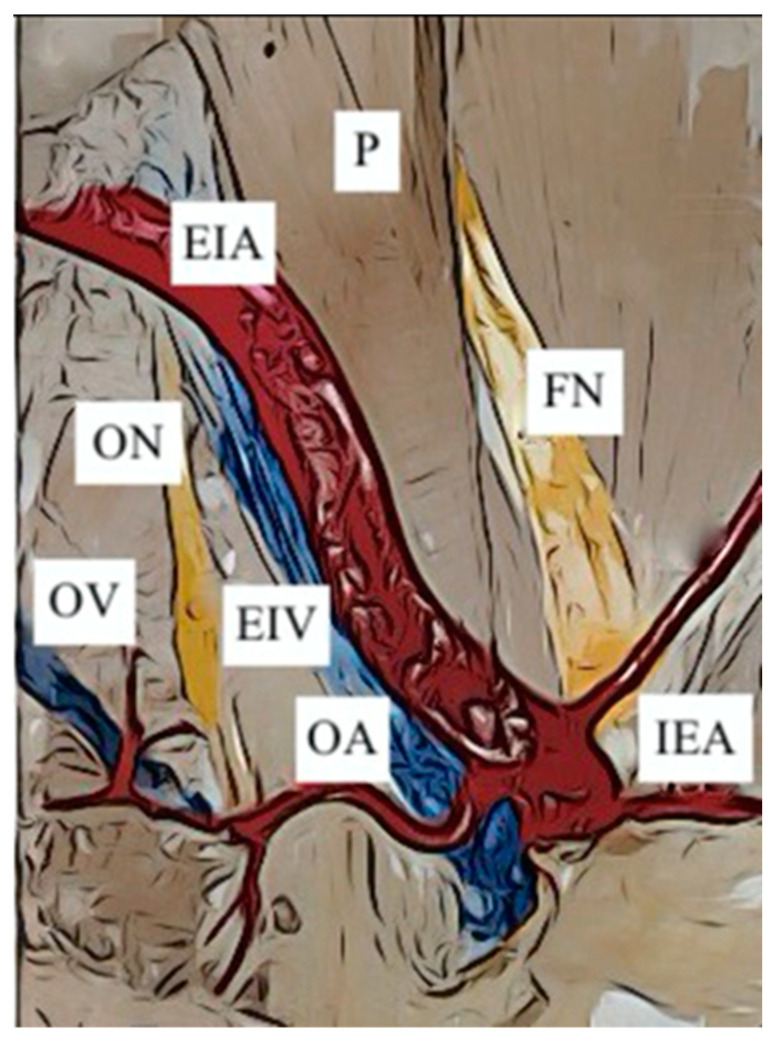
Type E. Obturator artery arising from the external iliac artery. EIA: external iliac artery; EIV: external iliac vein; IEA: inferior epigastric artery; IIA: internal iliac artery; OA: obturator artery; ON: obturator nerve; OV: obturator vein; P: psoas muscle; PT: posterior trunk of the internal iliac artery; RI: right iliac artery.

Origin of the OA from EIA. The origin of the OA from the EIA has been divided into only two subgroups, both already identified in the review of Sañudo 2011 (Table 4):

- Sañudo B (Figure 9): origin from the Inferior Epigastric Artery in 181 hemipelves (16.5%)
- Sañudo E (Figure 10): origin from the main trunk of the EIA in 62 hemipelvis (5.6%)

**Figure 11:**
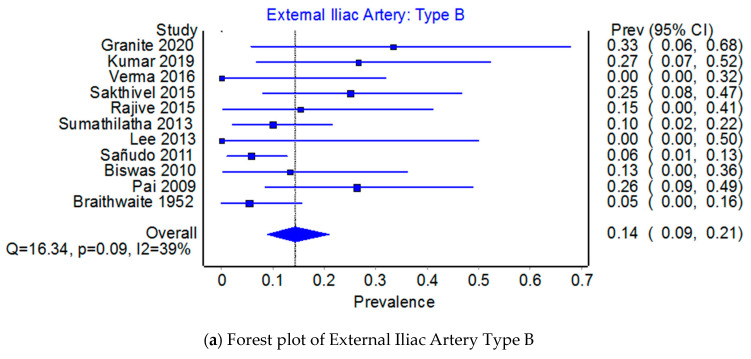

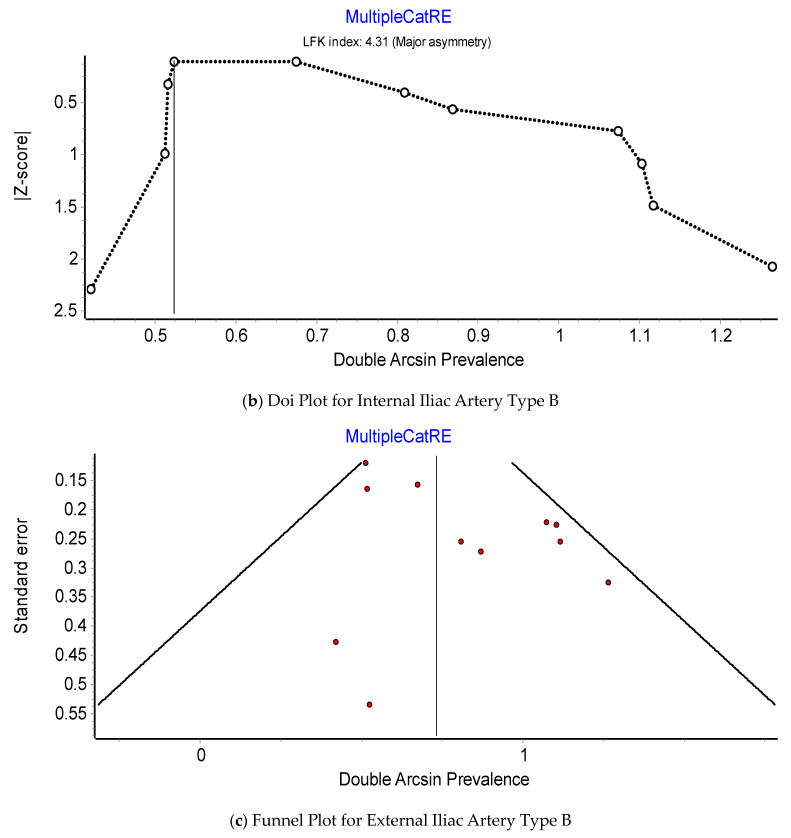
Prevalence of the External Iliac Artery: Type B.

**Figure 12:**
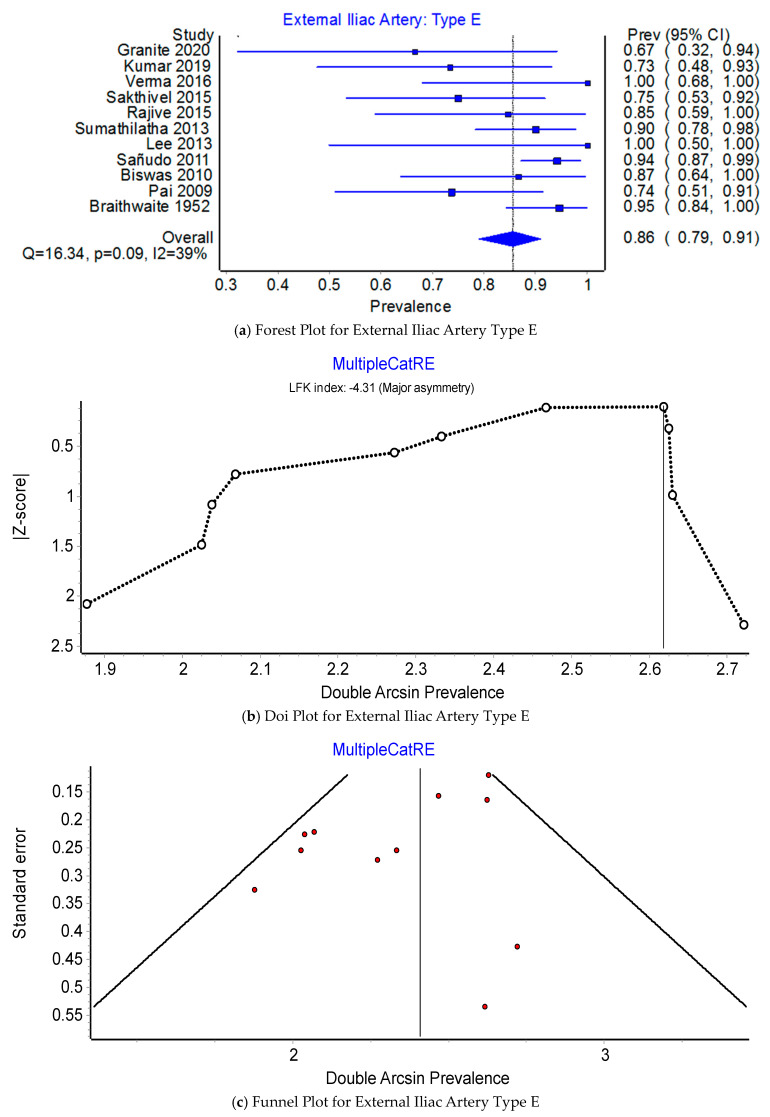
Prevalence of the External Iliac Artery: Type E.

Prevalence of the origin of the EIA: type B or type E. Across 11 studies that examined the origin of the EIA, the PPE for Type B was 85.6% (95% CI 79.0–81.1%) compared to 14.4% (95% CI 8.9–21.1%) for Type E (see Figure 11a and Figure 12a). The I^2^ heterogeneity was moderate at 38.8% (95% CI 0.0–69.9%), Q(1) = 16.33, *p* = 0.09, but exhibited major asymmetry as evidenced in the relatively empty areas of Doi plot and its LFK_index_ of ±4.31 (see Figure 11b,c). The leave-one-out PPEs range from 13.0% to 16.3% for Type E and 83.7% and 87.0% for Type B (see Figure 12b,c).

## 4. Discussion

In this systematic review and meta-analysis, the OA was reported in almost all cases (99.8%): the PPE for IIA was 77.7% (95% CI 71.8–83.1%) vs. 22.3% (95% CI 71.8–83.1%) for the EIA. In most cases, the OA originated from the anterior division trunk of the IIA (61.6%). As shown in the funnel and Doi plots, there was evidence of asymmetry, which could possibly be a result of publication bias/small effect bias; see Yurasakpong et al. (2021) for a discussion of this phenomenon in a similar anatomical case—the prevalence of the azygos lobe [14].

Variation of the OA origin is an important anatomical issue that has implications for a wide range of surgical procedures in different fields like gynecology, orthopedics, urology, vascular and oncological surgery. For instance, in the case of inguinal dissection (alone or in combination with an iliac/obturator lymph node dissection) for melanoma, identification, and control of the origin of the OA represents a critical step. Again, in cases of pelvic trauma, this considerable variation in origin may be a significant source of persistent bleeding that might be difficult to manage. The frequency with which vascular surgeons are called to treat groin complications is rapidly increasing also because of the widespread use of percutaneous procedures. Therefore, infected groin problems that often involve foreign prosthetic material or remnants of percutaneous femoral closure devices might be challenging and require control of bleeding, removal of foreign material, wide debridement, and sometimes arterial resection. Management of the consequential limb ischemia in such cases is controversial, and an obturator foramen bypass is a classical approach for the treatment of contemporary groin infection, thus requiring perfect knowledge of the OA course [15].

Surgery in the pelvic region must be conducted with extreme caution and with in-depth knowledge of possible vascular variations of the OA in particular.

The presence of an aberrant OA should be contemplated as a resource in case of IIA and its collateral branches are ligated or obstructed. An aberrant OA can therefore provide collateral circulation, especially in the area of the head of the femur.

The presence of an aberrant OA should be contemplated as a resource in case of IIA and its collateral branches are ligated or obstructed. An aberrant OA can therefore provide collateral circulation, especially in the region of the femoral head.

Thorough knowledge of the vascular pattern and awareness of potential arterial variations may decrease the risk of iatrogenic complications and may allow modification of the surgical/procedural approach to minimize this risk.

## 5. Conclusions

The variations in the origin of the obturator artery have been reviewed systematically and subjected to meta-analysis for the first time. A thorough understanding of pelvic vascular anatomy is fundamental in performing procedures like embolization, revascularization, treatment of pelvic fractures, surgery for advanced pelvic malignancy, and groin hernia surgery (both laparoscopic and open). A preoperative radiological/angiographic evaluation to know the pelvic vascular pattern and knowledge of obturator artery variants could help reduce the risk of iatrogenic injuries. It may also modify surgical strategy in order to minimize post-operative complications that can generate medical-legal disputes that are not easy to resolve.

## Figures and Tables

**Table 1:** Features of the included studies.

Author	Nation	Type of Evaluation	Number of Cadaveric Dissection
Granite 2020	USA	Cadaveric dissection	18
Kumar 2019	India	Cadaveric dissection	30
Verma 2016	India	Cadaveric dissection	27
Al-Talalwah 2016	United Kingdom	Cadaveric dissection	60
Sakthivel 2015	India	Cadaveric dissection	30
Rajive 2015	India	Cadaveric dissection	25
Lee 2013	Korea	Cadaveric dissection	18
Sumathilatha 2013	India	Cadaveric dissection	58
Sanudo 2011	United Kingdom	Cadaveric dissection	116
Jusoh 2010	Malaysia	Cadaveric dissection	17
Biswas 2010	India	Cadaveric dissection	28
Pai 2019	India	Cadaveric dissection	49
Braithwaite 1952	United Kingdom	Cadaveric dissection	85

**Table 2:** Origin of OA.

Author	Number of Hemipelvises Evaluated	Internal Iliac Artery (IIA)	External Iliac Artery (EIA)
Granite 2020	36	27 (75%)	9 (25%)
Kumar 2019	60	45 (75%)	15 (25%)
Verma 2016	54	49 (89.2 %)	5 (10.8%)
Al–Talalwah 2016	120	84 (70 %)	36 (30 %)
Sakthivel 2015	60	40 (66.7 %)	20 (33.3 %)
Rajive 2015	50	37 (74 %)	13 (26 %)
Lee 2013	34	31 (88.3 %)	3 (11.7 %)
Sumathilatha 2013	116	70 (60.3 %)	40 (34.5 %)
Sanudo 2011	224	155 (69.2 %)	69 (30.8%)
Jusoh 2010	34	34 (100 %)	0
Biswas 2010	56	41 (73.2 %)	15 (26.8 %)
Pai 2019	96	77 (80.2 %)	19 (19.8 %)
Braithwaite 1952	158	123 (77.8 %)	35 (22.2 %)

**Table 3:** The origin of the OA from the IIA.

Author	Origin from IIA	OA Branched from Main Trunk (Sanudo—Type d)	OA Branched from Anterior Trunk (Sanudo—Type a) (Normal)	Variation: OA Branched from Posterior Trunk (Common Trunk for Inferior Gluteal Artery and Internal Pudendal Artery) (Sanudo—Type c)
Granite 2020	27	2	22	3
Kumar 2019	45	0	24	21
Verma 2016	49	0	17	22
Al-Talalwah 2016	84	0	30	54
Sakthivel 2015	40	0	22	18
Rajive 2015	37	2	27	8
Sumathilatha 2013	70	0	36	34
Sanudo 2011	155	18	118	19
Jusoh 2010	34	0	32	2
Biswas 2010	41	0	25	16
Pai 2019	77	0	59	18
Braithwaite 1952	123	0	70	36

**Table 4:** The origin of the OA from the EIA.

Author	Origin from IEA	From Trunk of IEA (Sanudo—Type e)	From IEA (Sanudo—Type b)
Granite 2020	9	3	6
Kumar 2019	15	4	11
Verma 2016	5	0	5
Sakthivel 2015	20	5	15
Rajive 2015	13	2	11
Sumathilatha 2013	40	4	36
Lee 2013	3	0	3
Sanudo 2011	69	4	65
Biswas 2010	15	2	13
Pai 2019	19	5	14
Braithwaite 1952	35	33	2

## Data Availability

Not applicable.

## Abbreviations

OA	obturator artery
IIA	internal iliac artery
EIA	external iliac artery
